# Approaches and Vectors for Efficient Cochlear Gene Transfer in Adult Mouse Models

**DOI:** 10.3390/biom13010038

**Published:** 2022-12-26

**Authors:** Yu Zhao, Longlong Zhang, Daqi Wang, Bing Chen, Yilai Shu

**Affiliations:** 1 ENT Institute and Department of Otorhinolaryngology, Eye & ENT Hospital, State Key Laboratory of Medical Neurobiology and MOE Frontiers Center for Brain Science, Fudan University, Shanghai 200031, China; 2 Institutes of Biomedical Sciences, Fudan University, Shanghai 200032, China; 3 NHC Key Laboratory of Hearing Medicine, Fudan University, Shanghai 200031, China

**Keywords:** gene therapy, AAV, adult mouse, PSCC, RWM

## Abstract

Inner ear gene therapy using adeno-associated viral vectors (AAVs) in neonatal mice can alleviate hearing loss in mouse models of deafness. However, efficient and safe transgene delivery to the adult mouse cochlea is critical for the effectiveness of AAV-mediated therapy. Here, we examined three gene delivery approaches including posterior semicircular canal (PSCC) canalostomy, round window membrane (RWM) injection, and tubing-RWM+PSCC (t-RP) in adult mice. Transduction rates and survival rates of cochlear hair cells were analyzed, hearing function was recorded, AAV distribution in the sagittal brain sections was evaluated, and cochlear histopathologic images were appraised. We found that an injection volume of 1 μL AAV through the PSCC is safe and highly efficient and does not impair hearing function in adult mice, but local injection allows AAV vectors to spread slightly into the brain. We then tested five AAV serotypes (PHP.eB, IE, Anc80L65, AAV2, and PHP.s) in parallel and observed the most robust eGFP expression in inner hair cells, outer hair cells, and spiral ganglion neurons throughout the cochlea after AAV-Anc80L65 injection. Thus, PSCC-injected Anc80L65 provides a foundation for gene therapy in the adult cochlea and will facilitate the development of inner ear gene therapy.

## 1. Introduction

Hearing loss is the most common sensory deficit affecting humans today, disabling nearly 466 million people around the world [1]. The most common type of hearing loss is sensorineural hearing loss (SNHL), and this typically originates from damage to the delicate sensory structures or primary auditory neurons in the cochlea. There are two types of hair cells (HCs) within the cochlea, namely outer HCs (OHCs), which amplify sound, and inner HCs (IHCs), which transform sound waves into electrical signals that are transmitted to the brain via spiral ganglion neurons (SGNs).

Currently, treatment of SNHL is limited to using hearing devices that can amplify the sound, enhance sound transmission, or directly stimulate the neurons. Unfortunately, these methods are unable to resolve the limitations in hearing sensitivity and perception of natural sound in a noisy environment [2]. Thus, safer and more efficient strategies are an urgent need for the treatment of SNHL. The local injection of gene therapy agents into the inner ear is appealing. Conceptually, this allows a single administration of the gene therapy system to have a durable therapeutic effect. From the standpoint of safety, it overcomes the need for multiple injections in the hard-to-access cochlea, and the single administration offers a more stable environment for the sensitive cochlea. Over the last few decades, several studies have reported using intra-cochlear gene therapy to successful restore hearing in SNHL mouse models in which gene delivery was performed mainly during neonatal age [3,4,5,6]. Additionally, our group delivered therapeutic transgenes into the neonatal cochlea, and auditory function was effectively rescued [7,8,9,10,11,12,13]. However, one pivotal difference between human and mouse ears is the fact that the auditory system is fully mature at birth in human beings, whereas the mouse inner ear undergoes development and maturation until postnatal day (P) 16 [14,15], highlighting the need to develop a highly efficient gene therapy delivery strategy for the mature mouse ear. In addition, many SNHL cases are characterized by late-onset hearing impairment. For example, *GJB2, P2X2,* and *CDH23* mutations can manifest as delayed deafness [16,17], while *GSTMI, NAT, SOD2*, and *CAT* are associated with age-related hearing loss (ARHL) and noise-induced hearing loss (NIHL) [18,19,20]. Patients with mutations in these genes typically have onset in adulthood that might be beyond the reach of neonatal gene therapy, and thus, the application of gene therapy in adulthood is of vital significance. Moreover, compared with inherited factors, non-inherited forms of acquired SNHL account for a large proportion of the deaf population and are related to aging, noise, infection, and ototoxic drugs, all of which can lead to abnormal gene expression in the cochlea. A therapeutic gene editing system that targets genes with abnormal expression will offer a versatile, simple, efficient, and convenient treatment strategy to cure acquired non-inherited SNHL. We conclude that targeting the mature cochlea will improve on the current standard of care for both hereditary and acquired non-hereditary hearing loss.

Unlike neonatal mice, the bony labyrinth of the mature cochlea precludes the delivery of gene agents to the inner ear. To date, various gene delivery approaches have been examined. Among them, cochleostomy offers direct access to the scala media, but it has been shown to damage inner ear cells and to cause significant hearing loss [21]. Round window membrane (RWM) injection, with less surgical trauma than the cochleostomy approach [22], is recognized as a safe and clinically feasible method and has become the most common delivery method, while the results of RWM injection in the adult inner ear show diverse effects from transduction rates to hearing function [23,24,25]. Additionally, the canalostomy approach was explored for delivering gene therapy agents to the mature cochlea, which was recommended for use in adult mouse gene therapy because it provides a high transduction rate without causing loss of hearing function [26,27,28,29]. Furthermore, the trans-RWM injection combined with canal fenestration (RWM+CF) technique was recently developed and was reported to enable high-efficiency apex-to-base transduction while preserving hearing [24,30]. However, to our knowledge, there have only been limited reports evaluating the difference between these inner ear injection approaches in the adult cochlea.

In addition to the injection technique, a viral vector with high infection efficiency is also required. Different AAV serotypes have been successfully delivered into the neonatal mouse inner ear to target a wide range of cell types due to their safety and high efficiency. In brief, AAV-PHP.eB was reported to effectively transduce cochlear HCs and was used to deliver gene therapy systems into the inner ear to rescue auditory function in neonatal mice [8,9,10,11,31], AAV2 was injected via the RWM in neonatal mice and successfully delivered the transgene system to express otoferlin in a DFNB9 mouse model [4], and AAV-ie was found to transduce the cochlear supporting cells with high efficiency and to regenerate HC-like cells by delivering the transcription factor Atoh1 [32]. Additionally, AAV-Anc80L65 was shown to transduce cochlear HCs with high efficiency and to effectively rescue acquired SNHL in mice [7,33]. AAV-PHP.s was demonstrated to transduce peripheral neurons and deliver functional genes to control neuron excitability, but there are no reports of its use in the mouse inner ear [34,35]. Nevertheless, the cellular transduction efficiency in the inner ear might be different in neonatal and adult stages [24], and the transduction profiles of the above five recombinant AAVs have not been compared in the mature mouse cochlea.

In this study, we compared the delivery efficiency of three injection routes—posterior semicircular canal (PSCC) canalostomy, RWM, and tubing-RWM+PSCC (t-RP)—and five AAV types—AAV-PHP.eB, AAV-ie, AAV-Anc80L65, AAV2, and AAV-PHP.s—in the adult cochlea and showed that AAV serotype, injection volume, and delivery route all affected the final delivery efficiency. We concluded that 1 µL injection of AAV-Anc80L65 via the PSCC is a safe and reliable strategy for delivering gene therapy agents into the mature cochlea. Our findings will facilitate the development of gene therapy for hearing loss in adult animal models.

## 2. Materials and Methods

### 2.1. Animals

Adult C57BL/6J wild-type mice (4–6 weeks old) were used in this study. The mice were randomly assigned to the different experimental groups, with at least three mice in each group. All animals were housed in the Department of Laboratory Animal Science of Fudan University, and the animal experiments were approved by the Institutional Animal Care and Use Committee of Fudan University.

### 2.2. AAV Vector Construction 

AAV-PHP.eB, AAV-ie, AAV-Anc80L65, AAV2, and AAV-PHP.s were produced by the PackGene Biotech Company (Shanghai, China). All vectors were engineered to express the enhanced green fluorescent protein (eGFP) gene under the control of the chimeric cytomegalovirus (CMV) promoter and carried the woodchuck hepatitis post-transcriptional virus regulatory element (WPRE) cassette. To determine the transduction rate among the different serotypes, we adjusted all the vectors to be at equal doses of 1.0 × 10^13^ VG/mL. Virus aliquots were stored at −80 °C and thawed before use. 

### 2.3. Animal Surgery: PSCC Canalostomy

In brief, mice were anesthetized using an intraperitoneal injection of xylazine (10 mg/kg) and ketamine (100 mg/kg). Body temperature was maintained with a heating pad during the surgical procedure. The right post-auricular region was shaved and cleaned. A polyimide tube (0.1 mm diameter) was connected to a glass micropipette (504949, WPI, Sarasota, FL, USA) attached to a Nanoliter Microinjection System (NANOLITER2020, WPI Sarasota, FL, USA). Surgery was performed under an operating microscope. 

**Figure 1:**
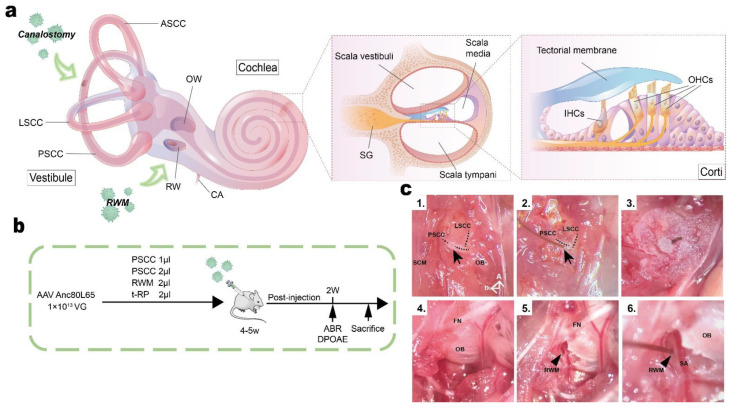
Overview of inner ear structures, the experimental design, and images showing the PSCC and RWM approaches. (**a**) Schematic diagram showing the anatomy of the inner ear and different surgical approaches. Green arrows show where the AAVs were injected. (**b**) The experimental timeline for delivery of AAV vectors with different surgical approaches. (**c**) Surgical pictures showing the PSCC (**c1**–**c3**) and RWM (**c4**–**c6**) approach. Black arrows indicate the hole in the PSCC before (**c1**) and after (**c2**,**c3**) the inoculation. Black arrowheads indicate the RWM location (**c5**) and the RWM inoculation (**c6**). PSCC: posterior semicircular canal, LSCC: lateral semicircular canal, ASCC: anterior semicircular canal, OW: oval window, RW: round window, CA: cochlear aqueduct, SG: spiral ganglion, SCM: sternocleidomastoid muscle, OB: otic bulla, FN: facial nerve, RWM: round window membrane, SA: stapedial artery, D: dorsal, A: anterior.

Canalostomy was first used to deliver AAVs into the mature cochlea through the PSCC approach [27,28]. A post-auricular incision was made using small scissors, and the sternocleidomastoid muscle (SCM) was divided to expose the PSCC. We then performed PSCC fenestration with a microprobe (Figure 1c1). The leakage of lymph confirmed successful access to the PSCC lumen. After the efflux abated (in less than 4 min), the tip of the polyimide tube was inserted into the hole through the fenestration site (Figure 1c2) and then sealed with tissue adhesive (3M Vetbond). For the two experimental groups, a total of 1 μL or 2 μL AAV vectors were injected into the cochlea through the polymide tube at a rate of 5 nL/s. After injection, the tubing was cut, and the injection tubing was left connected to the PSCC (~3 mm). The residual tubing was sealed by a muscle plug using tissue adhesive (Figure 1c3). The incision was closed with sutures and the mice were placed on a 42 °C heating pad for recovery. The total surgical time ranged from 20 to 30 min.

### 2.4. Animal Surgery: RWM Injection

After the post-auricular incision was made, the facial nerve was identified deep along the wall of the external auditory canal. A portion of the SCM was divided to expose the otic bulla ventral to the facial nerve (Figure 1c4). A hole was gently drilled in the otic bulla with a diameter 1–2 mm using an otologic drill, which was then widened sufficiently with forceps to visualize the stapedial artery and the RWM (Figure 1c5,c6). Next, the RWM was punctured gently with a glass pipette, and we observed fluid efflux through the RWM at this point. After the efflux had stabilized (less than 4 min), the tip of the polyimide tube was inserted into the RWM through the fenestration site (Figure 1c6), and a total of 2.0 μL AAV was then microinjected into the scala tympani at a rate of 5 nL/s. After pulling out the polyimide tube, the RWM niche was sealed quickly with a small plug of muscle to avoid leakage. The bony defect of the otic bulla was then sealed with small plugs of muscles using tissue adhesive. 6–0 nylon sutures were used to close the SCM and skin, and the mice were placed on a heating pad for recovery. Total surgical time ranged from 30 to 40 min.

### 2.5. Animal Surgery: Tubing-RWM+PSCC Injection (t-RP)

After exposing the facial nerve and the SCM, a portion of the muscle was divided to expose the otic bulla ventral to the facial nerve. An otologic drill was used to make a hole in the otic bulla and widen it sufficiently to visualize the stapedial artery and the RWM (Figure 1c5,c6). The PSCC was then exposed dorsal to the otic bulla, and a hole was drilled in the PSCC with a microprobe (Figure 1c1). Slow egress of the lymph confirmed a patent canalostomy, and we waited until the efflux stabilized. Again, it is important to remember that to minimize hearing loss, the lymph leakage time should be minimized after the fenestration [27]. Next, the RWM was gently punctured in the center, and 2.0 μL of AAV vectors were microinjected into the scala tympani through the polymide tube at a rate of 5 nL/s. After pulling out the tube, the RWM niche was quickly sealed with a small plug of muscle to avoid leakage. The bony defects of the otic bulla and canal were sealed with small plugs of muscles using tissue adhesive. The skin and the SCM were closed with 6-0 suture, and the mice were placed on a heating pad with bedding for recovery. Total surgical time ranged from 30 to 50 min. 

### 2.6. Hearing Tests 

Two weeks after injection, auditory brainstem responses (ABRs) and distortion product otoacoustic emissions (DPOAEs) were recorded in a soundproof chamber using the RZ6 Acoustic System (Tucker-Davis Technologies, Alachua, FL, USA). The signals were collected using subcutaneous needle electrodes inserted at the pinna (recording electrode), vertex (reference electrode), and rump (ground electrode) for ABR measurements. Closed-field ABR was recorded from mice anesthetized with xylazine (10 mg/kg) and ketamine (100 mg/kg) using an electret microphone (Electret Condenser) to record sound pressure in the ear canal. ABRs and DPOAEs were recorded during the same session.

Tone burst sound stimuli were presented at 4, 8, 16, 24, and 32 kHz to test the frequency-specific hearing thresholds. The sound level was decreased in 5 steps from 90 to 20 dB sound pressure level (SPL). ABR potentials were evoked and subsequently amplified 10,000 times with 1024 responses and bandpass filtered at 300 Hz–3 kHz at each SPL. The threshold of a certain frequency was determined as the lowest dB SPL at which any ABR wave could be detected upon visual inspection. The wave 1 amplitude was defined as the difference between the wave 1 peak and the average of the 1 ms pre-stimulus baseline. The mice were placed on a heating pad covered by a sterile drape to maintain their body temperature during the testing.

### 2.7. Fixation and Preparation of the Samples

Mice were briefly anesthetized with xylazine (10 mg/kg) and ketamine (100 mg/kg) in an isolated chamber and were transcardially perfused with ice-cold phosphate buffer solution (PBS) followed by 4% paraformaldehyde (PFA). Brains and injected and contralateral non-injected cochleae were rapidly extracted with a razor blade. All samples were stored in 4% PFA at 4 °C overnight. The brain samples were immersed in 30% sucrose in 1× PBS for 3 days until they sank, and they were then embedded in Tissue-Tek OCT compound before freezing in dry ice for 1 h and then sectioning into 10 μm slices using a cryostat (Leica Biosystems, San Diego, CA, USA). The cochlear samples were decalcified with 10% EDTA at 4 °C for at least 3 days. 

### 2.8. Immunohistochemistry and Confocal Microscopy

For animals that were used to explore the cell tropism and transduction efficiency of different AAV serotypes, injected and contralateral non-injected cochleae were harvested after the animals were sacrificed by cervical dislocation. Samples were immersed in 4% PFA at 4 °C overnight followed by decalcification with 10% EDTA at 4 °C for 1–3 days. For whole-mount immunofluorescence staining, the decalcified cochleae were dissected in PBS into three pieces designated as the apical, middle, and basal turns. For frozen sections, serial cryostat sectioning of the cochleae embedded in OCT into 9 μm sections was performed on an ultramicrotome (LKB 8800 Ultrotome III) after gradient dehydration in sucrose solutions (15% sucrose, 30% sucrose, OCT). The tissues were blocked in 1% Triton X-100 in PBS with 10% donkey serum at 4 °C overnight and then incubated with rabbit polyclonal Myosin7a (1:500 dilution, Proteus Biosciences, Ramona, CA, USA) and mouse-anti TUJ1 (1:500 dilution, Biolegend, San Diego, CA, USA) primary antibodies. Fluorescence-labeled donkey anti-rabbit IgG Alexa Fluor 647 and goat anti-mouse IgG2a Alexa Fluor 555 (1:500 dilution, Thermo Fisher Scientific, MA, USA) secondary antibodies were incubated in the dark for 2 h at room temperature after rinsing three times with PBS. Samples were mounted in ProLong Diamond Antifade Mountant with DAPI (#P36962, Thermo Fisher Scientific, MA, USA) and observed with a Leica TCS SP8 confocal microscope (Leica Microsystems Inc., Bannockburn, IL, USA). Images were acquired in a 1024 × 1024 raster with 1–2 μm z-steps for whole-mount samples and 0.5 μm z-steps for frozen section samples. 

### 2.9. Hematoxylin and Eosin (H&E) Staining 

To explore the cochlear morphology, the cochlear sections were stained with H&E according to standard protocols. 

### 2.10. Cell Counting 

ImageJ was used to acquire maximum intensity projections of z-stacks for each segment, and Adobe Photoshop was used to merge the images. For HC counting, the numbers of Myo7a+HCs and the numbers of eGFP+/Myo7a+ cells in the sensory epithelium in whole-mount samples were counted in every 100 μm region of the apical, middle, and basal turns of the cochlea. The outer three rows of cells were OHCs and the inner single row was IHCs. Segments with dissection-related damage were omitted from the analysis. 

### 2.11. Statistical Analyses

Data are expressed as the mean ± SEM. Statistical analyses were performed with GraphPad Prism 9 (San Diego, CA, USA). We used two-way ANOVA with Bonferroni corrections for multiple comparisons for virus transduction efficiencies, for the quantification of HC survival, and for ABR tests. The level of significance was set at *p* < 0.05.

## 3. Results

### 3.1. In Vivo Delivery of AAV-Anc80L65 into Adult Cochleae via Different Injection Routes

To compare the effect of the injection route on inner ear transduction rates and hearing in adult mice, we selected the AAV-Anc80L65 vector due to its high transduction efficiency and used the PSCC canalostomy, RWM injection, and t-RP approaches (Figure 1a,b). For PSCC canalostomy, successful opening of the bony canal was assessed by observing lymphatic leakage, allowing to the insertion of the injection tube (Figure 1c1,c2). Because of the small size of the structures, it was impossible to determine whether the viral suspension was administered into the endolymph or the perilymph. In our experiments, the microprobe drilled vertically until we observed lymphatic leakage from the bony defect of the PSCC. Then, the tip of the microprobe was changed for an oblique orientation, more tangent to the canal, towards the ampulla, before being drawn. The RWM approach is used for cochlear implantation in humans and appears to be a safe and clinically feasible route. However, in adult mice a special physiological structure, the ossified otic bulla, blocks access to the RWM (Figure 1c4). It is, therefore, notable that the first step in RWM injection in adult mice is drilling a small hole in the otic bulla (Figure 1c5). We recommend that the hole should be drilled as accurately as possible and of the right size, and it is important not to touch the stapedial artery (Figure 1c6). For the t-RP route, different from the reports by Yoshimura et al. [24], we used a tube connecting to a glass micropipette to perform the whole surgery. To make sure the time of lymphatic efflux was shorter than 5 min [27], the RWM and stapedial artery are recommended to be exposed first, and the next step is to locate the PSCC and drill a hole in it (supplementary Figure S1a). Again, the hole should be deep enough to ensure the puncture of the lymphatic space. A total of 1 μL trypan blue was used to test the perfusion of the surgery. We observed that the cochlea turned blue and the dye overflowed from the PSCC fenestration site and RWM inoculation site (supplementary Figure S1b,c), an indication that the surgery allows an effective perfusion of flow pattern. Importantly, the tube and glass micropipette need to be disinfected before use.

### 3.2. Canalostomy Resulted in Robust Transduction of HCs throughout the Cochlea

**Figure 2:**
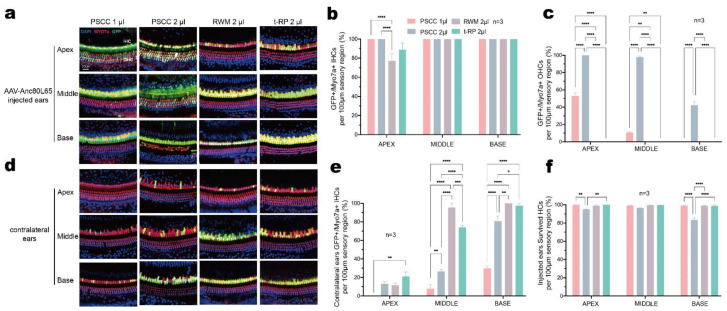
The AAV delivery approaches make a difference in HC transduction and HC survival in the adult cochlea. The cochleae were harvested 2 weeks after AAV-Anc80L65 was delivered into the adult cochlea (4–6 weeks old). HCs were labeled with Myo7a (red), the nuclei were stained with DAPI (blue), and native eGFP was observed (green). (**a**,**d**) Representative high-magnification images of the apex, middle, and base in the AAV-Anc80L65 (1.0 × 10^13^ VG/mL)-injected ears (**a**) and contralateral ears (**d**). (**b**,**c**) Quantitative comparison of IHC transduction efficiency (**b**) and OHC transduction efficiency (**c**) in injected ears in the four different groups as assessed in 100 μm segments across different regions of the cochlea (apex, middle, and base). (**e**) Quantitative comparison of IHC transduction efficiency in the contralateral ears in the four different groups, as assessed in 100 μm segments across different regions of the cochlea (apex, middle, and base). (**f**) Quantification of HCs survival at 2 weeks after AAV-Anc80L65 injection via the different approaches in adult mice, as assessed in 100 μm segments across different regions of the cochlea (apex, middle, and base). Two-way ANOVA with Bonferroni correction was performed for multiple comparisons of transduction rates and survival rates. * *p* < 0.05, ** *p* < 0.01, *** *p* < 0.001, **** *p* < 0.0001.

We first injected different volumes of Anc80L65 into the inner ear of 5-week-old mice via the PSCC approach and analyzed the transduction efficiency by immunohistochemistry after 2 weeks. The injection of Anc80L65 using the PSCC approach led to robust transduction in both IHCs and OHCs (Figure 2a,d). For the group injected with 1 μL, the transduction rates for IHCs and OHCs were 100% and 52.81 ± 3.42% in the apical turn, 100% and 10.49 ± 0.85% in the middle turn, and 100% and 0% in the basal turn of the cochlea, respectively (Figure 2a–c and Figure S2). For the group injected with 2 μL, we observed a more robust transgene expression in HCs throughout the cochlea, and the transduction rate was 100% in the apical turn for both IHCs and OHCs, 100% and 97.44 ± 1.48% in the middle turn, and 100% and 42.02 ± 4.48% in the basal turn, respectively (Figure 2a–c). These immunofluorescence results imply that the transduction efficiency is dependent on the volume of AAV injection.

To avoid the difference in inner ear transduction by the virus as much as possible, we delivered Anc80L65 into adult cochleae with the same AAV titer and volume (1.0 × 10^13^ VG/mL) via the RWM and t-RP approaches. RWM injection in mice was reported to be well-tolerated at larger volumes of 2 μL [36]. We found a variable transduction efficiency across tonotopic positions for IHCs (RWM group: apex 77.02 ± 7.53%, middle 100%, base 100%; t-RP group: apex 88.64 ± 7.31%, middle 100%, base 100%) (Figure 2a,b). No eGFP expression was detected in OHCs in these two groups (Figure 2a,c). We concluded that AAV transduction efficiency is dependent on the injection volume and that PSCC canalostomy leads to a higher transduction rate in both cochlear IHCs and OHCs.

In our previous study, we found that when AAVs were injected into one side of the cochlea in neonatal mice, the HCs were also successfully transduced in the contralateral ears [6,12]. Surprisingly, we also observed transgene expression in the contralateral HCs in adult mice (Figure 2d). The injection of Anc80L65 in 5-week-old mice using the above four methods resulted in variable transduction of IHCs throughout the contralateral cochlea. In the 1 μL PSCC group, the transduction rates for IHCs were 0% in the apex, 7.69 ± 4.44% in the middle turn, and 29.7 ± 2.46% in the base. For the 2 μL PSCC group, the transduction rates of IHCs were more robust and were 13.03 ± 2.35% in the apex, 26.28 ± 2.31% in the middle, and 80.56 ± 5.56% in the base, suggesting that the transduction efficiency in the contralateral ears is also dependent on the volume of AAV injection. At the same time, eGFP expression in the contralateral IHCs was also observed with the other injection approaches. In the RWM group, the transduction rate was 11.36 ± 2.66% in the apex, 95.24 ± 4.76% in the middle, and 100% in the base, and in the t-RP group, the transduction rate was 20.94 ± 4.93% in the apex, 73.72 ± 2.31% in the middle, and 97.22 ± 2.78% in the base, demonstrating that the AAVs could migrate from the injected to the non-injected side after the initial injection via all tested surgical routes, but especially for the RWM and t-RP groups (Figure 2e). Notably, all contralateral IHCs had a higher transduction rate in the basal turns compared to the corresponding apical and middle turns, whereas no OHC transduction was observed (Figure 2d,e). Together, our results imply that the transgene expression in the contralateral HCs is dependent on the volume of injected AAVs and on the surgical method. 

### 3.3. Preservation of Hearing Function via PSCC Canalostomy in Adult Mice

To compare the impact of injection approaches on auditory function, at 2 weeks after injection, we calculated HC survival by immunohistochemistry and assessed ABRs and DPOAEs. Interestingly, HC loss was observed in cochleae injected with 2 μL Anc80L65 after canalostomy, especially in the basal turn, mainly in OHCs (Figure 2a,f and Figure S3). To investigate the difference in HC loss, we quantified HC survival in Anc80L65-injected ears in whole-mount preparations along the entire extent of the cochlear duct. In the injected cochlea, the HC survival rates were 94.82 ± 0.59% in the apical turn, 96.47 ± 0.99% in the middle turn, and 83.32 ± 2.35% in the basal turn. At the same time, no evidence of obvious HC death (<3%) was observed in the 1 μL PSCC, RWM, or t-RP groups in the three turns (Figure 2f and Figure S3). These analyses suggest that injecting a total of 2 μL AAV vector via the PSCC approach will likely impair HCs, and an injection volume of 1 μL is thus optimal for canalostomy in mature cochlear viral delivery. 

**Figure 3:**
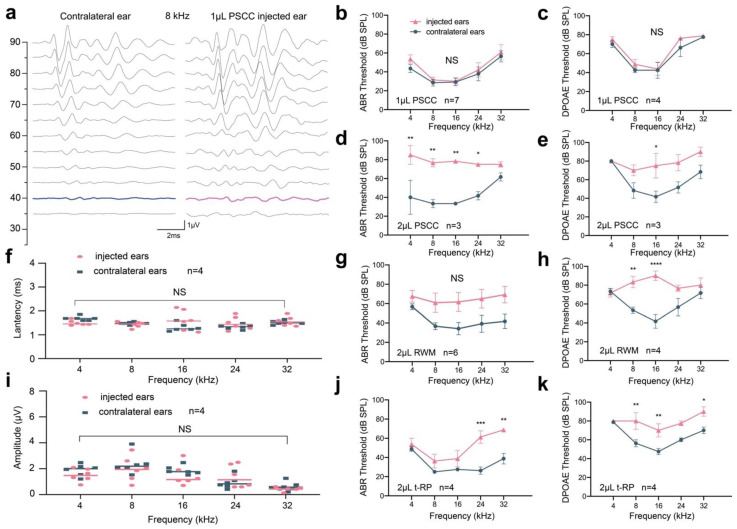
Cochlear injection of 1 μL AAV-Anc80L65 via PSCC canalostomy in adult mice (4–6 weeks old) does not impair hearing function. ABRs and DPOAEs were recorded at 2 weeks after the injection. (**a**) ABR waveforms from the representative contralateral ear and the ear injected with 1 μL AAV-Anc80L65 vector through the PSCC approach at 8 kHz. The blue trace indicates the threshold of the contralateral ear while the pink trace indicates the threshold of the injected ear. The scale bar applies to all traces. (**f**,**i**) Latencies (**f**) and peak amplitudes (**i**) of ABR wave 1 evoked by 90 dB SPL at 4, 8, 16, 24, and 32 kHz in the AAV-Anc80L65-injected ears compared with the contralateral ears 2 weeks after 1 μL PSCC injection. (**b**–**e**,**g**,**h**,**j**,**k**) ABR and DPOAE tests showed no threshold shifts in the 1 μL PSCC group inner ears compared with the contralateral non-injected control ears, whereas the hearing threshold increased at different frequencies to different degrees in the remaining groups. Two-way ANOVA with Bonferroni correction was performed for multiple comparisons for ABR and DPOAE tests. * *p* < 0.05; ** *p* < 0.01; *** *p* < 0.001; **** *p* < 0.0001.

Although we saw an increase of 10 dB in the ABR threshold at 4 kHz, we observed no statistically significant differences in tone-burst ABRs or DPOAEs across all frequencies between ears for the 1 μL PSCC group (Figure 3a–c). We also observed no significant changes in amplitudes or latencies in ABR wave I (Figure 3f,i), indicating that using the PSCC approach to deliver 1 μL AAV into the adult cochlea could preserve auditory function, and there was no significant damage to mechanoelectrical transduction in sensory cells anywhere along the cochlear spiral. However, in the 2 μL PSCC group, we found a significantly elevated ABR and DPOAE threshold shift across all frequencies as compared to the contralateral ears (Figure 3d,e). In detail, the ABR thresholds in the injected ears were increased by 30 dB at 4 kHz, 43.33 dB at 8 kHz, 45 dB at 16 kHz, 34 dB at 24 kHz, and 13.33 dB at 32 kHz (Figure 3d), and the DPOAE thresholds in injected ears were increased by 0 dB at 4 kHz, 21.67 dB at 8 kHz, 45 dB at 16 kHz, 34 dB at 24 kHz, and 13.33 dB at 32 kHz (Figure 3e). HC loss may be one of the reasons for the hearing impairment (Figure 2f and Figure S3). In the RWM group, we also observed increased ABR thresholds of 10.83 dB at 4 kHz, 24.17 dB at 8 kHz, 27.50 dB at 16 kHz, 25.83 dB at 24 kHz, and 27.50 dB at 32 kHz, but these shifts were not statistically significant (*p* > 0.05) (Figure 3g). The DPOAE thresholds in the RWM group were also significantly increased by 30 dB at 8 kHz and 48.33 dB at 16 kHz (Figure 3h). Similarly, in the t-RP group, an elevation in ABR was detected at 24 and 32 kHz. Increased shifts of DPOAE thresholds were also detected (Figure 3j,k).

To further evaluate the difference in hearing function in each group, we analyzed all of the hearing results together (supplementary Figure S4). We found statistically significant differences in ABR measurements between the 1 μL PSCC group and 2 μL PSCC group at 4, 8, and 16 kHz and between the 1 μL PSCC group and RWM group at 8 and 16 kHz. Statistically significant differences in DPOAEs were also recorded between the 1 μL PSCC group and 2 μL PSCC group at 8 and 16 kHz, between the 1 μL PSCC group and RWM group at 16 kHz, and between the 1 μL PSCC group and t-RP group at 8 and 16 kHz. Again, these results demonstrated that an injection volume of 1 μL AAV through the PSCC is highly efficient and does not impair hearing function in the mature rodent cochlea. Based on these results, it is concluded that adult injection of AAVs by PSCC canalostomy does not significantly affect normal hearing, further supporting that 1 μL of AAVs can be safely administered via canalostomy using the PSCC approach.

### 3.4. Local Injection in the Inner Ear Allows AAV Vectors to Spread to the Brain

**Figure 4:**
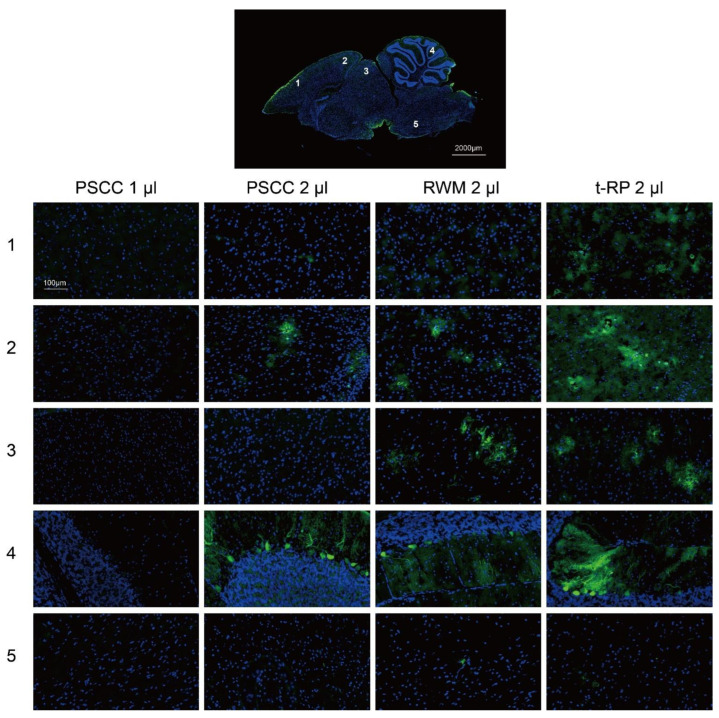
Frozen sagittal brain sections from AAV-Anc80L65-injected adult mice (4–6 weeks old) using different surgical approaches. Two weeks after the cochlear injection, brain tissues were rapidly extracted. The whole sagittal brain section image at the top came from the t-RP group, and the high-magnification images were taken from the following brain regions of each group: the cerebral cortex (1 and 2), midbrain (3), cerebellum (4), and hindbrain (5). The cell nuclei were stained with DAPI (blue), and native eGFP was imaged (green).

To explore the relationship between cochlear injection approaches and brain gene transfer in rodents, we examined the eGFP expression in mouse brains (Figure 4). The results showed that the t-RP approach led to wider transduction in brains, including the cerebral cortex, midbrain, and cerebellum, and neither surgical wound infection nor tympanitis were observed 2 weeks after the surgery, an indication that this is an appropriate surgical procedure. It is, thus, concluded that the delivery method will influence the distribution of AAV vectors in rodents. 

### 3.5. AAV-Anc80L65 Showed More Efficient Transduction in Cochlear HCs and SGNs

**Figure 5:**
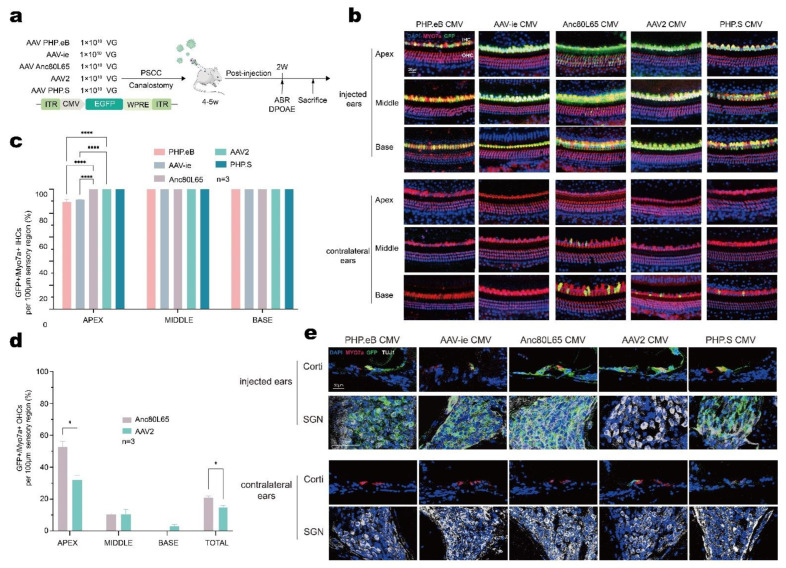
In vivo transduction of cochlear cells with AAV-PHP.eB, AAV-ie, Anc80L65, AAV2, and PHP.s in adult mice (4–6 weeks old). (**a**) Experimental design of the in vivo cochlear transduction. (**b**) Representative high-magnification images of the apex, middle, and base with different AAV vectors (1.0 × 10^13^ VG/mL). (**c**,**d**) Quantitative comparison of IHC transduction efficiency (**c**) and OHC transduction efficiency (d) in the injected ears assessed in 100 μm segments across different regions of the cochlea (apex, middle, and base). (**e**) Representative images of the organ of Corti and TUJ1-positive SGNs. Two-way ANOVA with Bonferroni correction was performed for multiple comparisons for cochlear HC transduction. * *p* < 0.05; **** *p* < 0.0001.

Because the injected volume of 1 μL AAV through the PSCC was shown to be a safe and efficient method, we used this method to assess the cell tropism and transduction efficiency of different AAV serotypes. Five AAV vectors of different serotypes (AAV-PHP.eB, AAV-ie, AAV-Anc80L65, AAV2, and AAV-PHP.s) were adjusted at equal doses of 1.0 × 10^13^ VG/mL, then 1 μL of the AAV vectors were injected into the adult cochlea via the PSCC (Figure 5a). 

According to the immunofluorescence results, one row of IHCs and three rows of OHCs were arrayed regularly across the entire length of the cochlea in both ears, an indication that no major HC death was induced by adult cochlea injection or AAV infection (Figure 5b). Immunostaining results showed that all five AAV serotypes yielded eGFP-positive IHCs in the cochlea with robust transduction rates varying from 89.32% to 100%. PHP.eB injection showed the lowest transduction in the apical turn (89.32 ± 2.35%) (Figure 5b,c). AAV-ie injection also showed a less efficient transduction rate in the apex (91.16 ± 0.25%) (Figure 5b,c). Anc80L65 and AAV2 were capable of infecting some but not all OHCs (Anc80L65: apex 52.81 ± 3.42%, middle 10.49 ± 0.85%, and base 0%; AAV2: apex 32.20 ± 2.63%, middle 10.73 ± 2.98%, and base 15.23 ± 0.79%) (Figure 5d). To evaluate the transduction efficiency in OHCs, we analyzed the total eGFP-positive OHCs and found that compared with AAV2 Anc80L65 provided more effective gene transfer (*p* = 0.033). In the contralateral ears, we also observed sporadic gene transfer from the basal to middle turn after injection with Anc80L65, AAV2, and PHP.s (Figure 5b). Together, the transduction analysis demonstrated higher eGFP intensity in the apex and lower eGFP intensity in the base for the injected ears, while in contralateral ears, increased eGFP intensity was found only in the base. To evaluate the cell tropism, we then labeled SGNs with TUJ1 and analyzed the eGFP expression in SGNs (Figure 5e). We found that all AAV serotypes provided similar transduction in SGNs except for AAV2, which showed no significant gene transfer. These results suggest that AAV serotypes affect inner ear gene delivery and that cell tropism varies with different AAV serotypes. For the adult cochlea, Anc80L65 showed higher efficiency in transducing cochlear HCs.

## 4. Discussion

In this study, we focused on the fully developed cochlea and investigated different delivery approaches and different AAV serotypes for their specificities of cochlear cell-type targeting in the mouse inner ear. Our results demonstrated that delivery of 1 μL AAV via PSCC canalostomy is efficient and does not impair hearing functions in the mature rodent cochlea, and we showed that Anc80L65 is among the most efficient for delivering genes primarily into auditory HCs and SGNs.

AAV vectors are emerging as attractive vehicles for inner ear gene therapy due to their satisfactory safety profile and their ability to transduce various cell types in the cochlea [3,4,5,7,8,12,32,37]. Despite the extensive information using various AAV serotypes in the neonatal cochlea, there are limited and variable pieces of information available about AAV gene therapy in the fully-developed mature cochlea. The major reason for this is the technically challenging induction in the target tissue, the organ of Corti, which is exceptionally delicate and is encased in a protective bony labyrinth, thus limiting the types of surgical approaches that can be used. Various cochlear delivery approaches have been examined, including cochleostomy, canalostomy, RWM injection, and RWM+CF procedures for inner ear gene delivery. Compared with the RWM injection or canalostomy approach, which adjoin the perilymph, AAVs can be injected via cochleostomy into the endolymph from the scala media where HCs are located. However, several cochleostomy studies have demonstrated inevitable cochlear damage and highly variable transduction efficiency [21,22,38]. Thus, we excluded the cochleostomy methods to deliver AAVs into the mature cochlea.

Consistent with previous findings [22,23,24], RWM injection performed in adult mice showed an apex to base gradient in HC transduction in the injected ears (Figure 2a,b). In addition, the intervention itself causes only mild auditory damage [24,25], which was confirmed in our results (Figure 3g,h). At the same time, we found a similar elevation in HC transduction from the apex to the base throughout the cochlea with limited transduction in the apical turn after the t-RP procedure (Figure 2a,b), and auditory threshold shifts were also observed (Figure 3j,k). We suggest the following reasons for these observations. (1) The RWM injection and t-RP procedures need to open the RWM, which cannot be blocked after the inoculation, and this will lead to fluid leakage from the fenestra and decrease the volume of injected-AAV vectors. (2) The otic bulla is completely ossified in adult mice [14], making access to the RWM much more challenging and potentially more traumatic. (3) To visualize the RWM, a hole must be drilled in the ossified otic bulla, which may induce middle ear effusion [25], which negatively affects hearing. (4) The highly sensitive mechanosensory cochlear tissue is vulnerable to both the pressure and volume requirements associated with RWM injection techniques [24].

Similarly, we observed efficient transduction in inner ears without hearing impairment when the PSCC route was used to deliver AAVs [28,29,38]. Nevertheless, the transduction rates were diverse in cochlear HCs compared with some reports. For example, Kang et al. showed that Anc80L65 transduction via PSCC injection was only seen in IHCs [39], whereas we observed robust eGFP expression in all IHCs and some OHCs (Figure 2a and Figure S2). The dose, titer, and transgene design of AAVs may lead to distinct differences in IHC and OHC transduction efficiency. The AAV we used had higher titers and carried the WPRE cassette, both of which could increase eGFP expression levels [24,40]. In addition, the manufacturer of the virus was different, which may have led to differences in viral purification and processing, further impacting GFP expression [41]. In a previous study, the volume of 2 μL is well-tolerant for RWM injection in P10–12 mice [36]; therefore, we tried to inject a total volume of 2 μL AAV vectors into adult cochlea. However, we recorded an elevated hearing threshold in the 2 μL PSCC group (Figure 3d,e), which was in accordance with another conclusion [28]. Moreover, we observed the HCs loss in the 2 μL PSCC group. As the adult cochea is highly vulnerable, the sound sensing structures was delicate, including HCs, the cilia of HCs, auditory neurons and/or nerves. Thus, we reasoned that the cause of hearing impairment in the 2 μL-PSCC group may including but not limited to HCs loss.

Previously, Suzuki et al. showed that Anc80L65 injection via the PSCC in adult mice resulted in the transduction of all IHCs, and the OHC targeting tended to increase from base to apex [29]. Zhu et al. used the surgical technique for PSCC gene delivery, but they only investigated one AAV serotype, and that AAV serotype was shown to influence transduction efficiency in the inner ear [24]. To identify safe and efficient transduction of AAV vectors into the adult mouse inner ear, we selected five AAV serotypes—including AAV-PHP.eB, AAV-ie, AAV-Anc80L65, AAV2, and AAV-PHP.s—for delivery into the adult cochlea via the PSCC (Figure 5a). All AAV serotypes examined in this study infected mature IHCs, but the infection efficiency was lower at the apex with PHP.eB and AAV-ie (Figure 5b,c). Interestingly, we found that all but AAV2 transduced SGNs, despite robust transduction being observed in cochlear HCs (Figure 5e), suggesting that AAV2 tropism is more specific than that of other AAVs. 

Another important aspect of the current study was the distributions of AAV vectors after trans-cochlear injection. Numerous gene delivery studies have focused on neonatal mice. In previous mouse studies, a patent cochlear aqueduct was observed and local AAV injection through the RWM in P0–P2 mice resulted in the transduction of brain cells [33]. Our study detected slight eGFP expression in the contralateral non-injected ear, with decreased transduction from the base to the apex, and slight eGFP expression in brain. A possible reason for this unintended transduction is that AAV migrated from the injected cochlea to the contralateral non-injected cochlea mainly through the cochlear aqueduct [42,43]. Interestingly, the eGFP expression of the “unintended transduction” was diverse from surgical approach to AAV serotypes. Thus, we reason that the regional off-target transduction might due to differences in AAV tropism and the cochlear injection route. Although the biological barriers in the mature inner ear, including the blood-labyrinth barrier and tissue barriers, do not preclude the possibility of AAV vectors reaching the CNS after cochlear delivery, the robust transgene transduction was detected mainly in the injected cochlea. In addition, we observed the ABR thresholds in the contralateral ears at 32 kHz were different between PSCC, RWM, and t-RP groups, although there exist no statistically significant differences (Figure 3b,d,g,j). As the mice comes from the same strain, C57BL/6J, and the age of mice were 4–6 weeks when the surgery was performed, we reasoned that the elevated ABR thresholds may largely come from individual difference, and the sample size should be enlarged to clarify the hearing of high frequency after the surgery. On all accounts, regional off-target transduction to the contralateral ears or brain via unilateral inner ear injection may lead to unintended results, and further studies should be conducted to evaluate the security of inner ear injection, including whole-body gene transfer test, vestibular functional evaluations, brain functional test, and immunological assays. 

Taken together, our results demonstrate that PSCC-injected Anc80L65 provides the highest auditory efficiency of the AAV serotypes we tested. These findings open the door to further evaluation of therapeutic gene transfer for forms of hearing loss affecting HCs and SGNs.

## Data Availability

As a rule of our laboratory, the datasets used in the current study are available from the first author (zy_199301@163.com) on reasonable request.

## Supplementary Materials

The following supporting information can be downloaded at: https://www.mdpi.com/article/10.3390/biom13010038/s1, Figure S1: Surgical picture of trypan blue injection in adult cochlea. Figure S2: Representative high-magnification images of the apex, middle, and base for the PSCC group injected with 1 μL AAV-Anc80L65 (1.0 × 10^13^ VG/mL). Figure S3: Quantification of IHCs and OHCs survival at 2 weeks after AAV-Anc80L65 injection via the different approaches in adult mice, as assessed in 100 μm segments across different regions of the cochlea (apex, middle, and base). Figure S4: The hearing results involving all groups at 2 weeks after the Anc80L65 injection in adult mice.
