# Supplementary material for: Approaches and Vectors for Efficient Cochlear Gene Transfer in Adult Mouse Models
Source: Biomolecules. 2022 Dec 26;13(1):38. doi: 10.3390/biom13010038 (PMC9855574; doi:10.3390/biom13010038)
Supplement: Supplementary file 1 [file biomolecules-13-00038-s001.zip › biomolecules-2014937-supplementary.pdf]

SUPPLEMENTAL MATERIALS

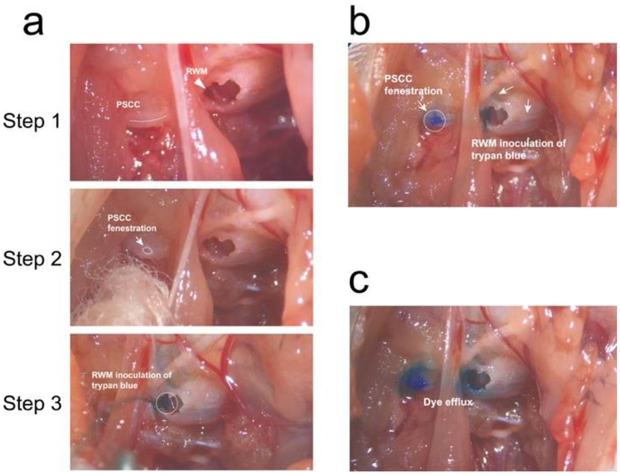

Figure S1. Surgical picture of trypan blue injection in adult cochlea. (a) Surgical procedures of t-RP approach. White arrows indicate the hole in the PSCC, white arrowhead indicates the location of RWM, white circles indicate the leakage of injected drugs. (b) The cochlea turned blue and the dye were efflux from the PSCC fenestration site, as the white arrow indicates. (c) After we finished the inoculation and draw back the injected tube, the obvious leakage of injected dye was observed. (OB: otic bulla, RWM: round window membrane, PSCC: posterior semicircular canal.)

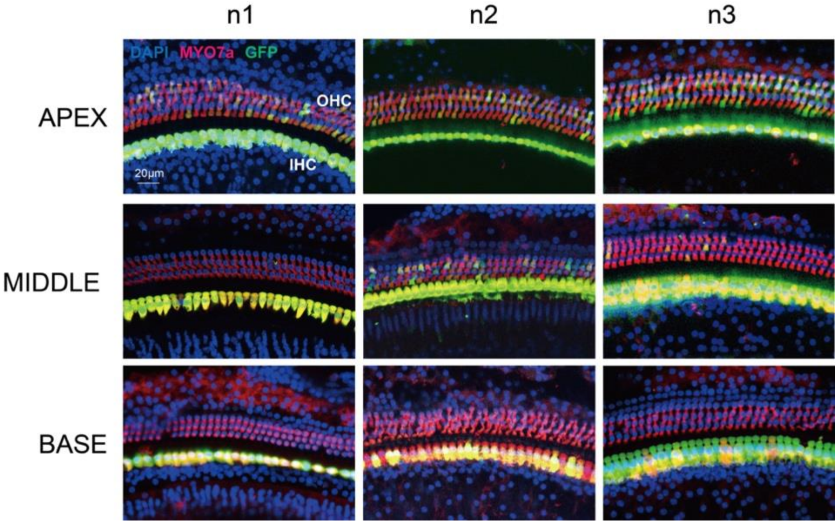

Figure S2. Representative high-magnification images of the apex, middle, and base for the PSCC group injected with 1 μL AAV-Anc80L65 (1.0×10^{13} VG/ml). n1, n2, n3 represents three different samples.

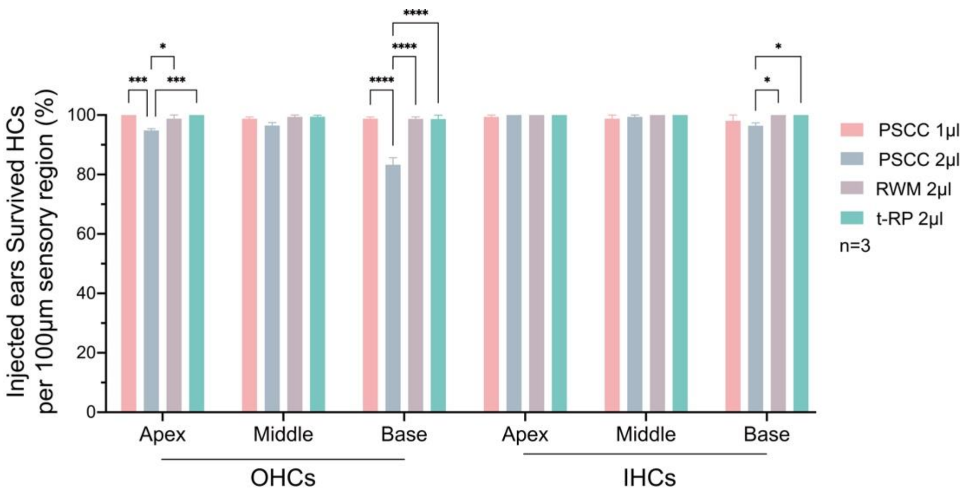

Figure S3. Quantification of IHCs and OHCs survival at 2 weeks after AAV-Anc80L65 injection via the different approaches in adult mice, as assessed in 100 μm segments across different regions of the cochlea (apex, middle, and base). Two-way ANOVA with Bonferroni correction was performed for multiple comparisons of transduction rates and survival rates. **p < 0.05, ***p < 0.001, ****p < 0.0001.*

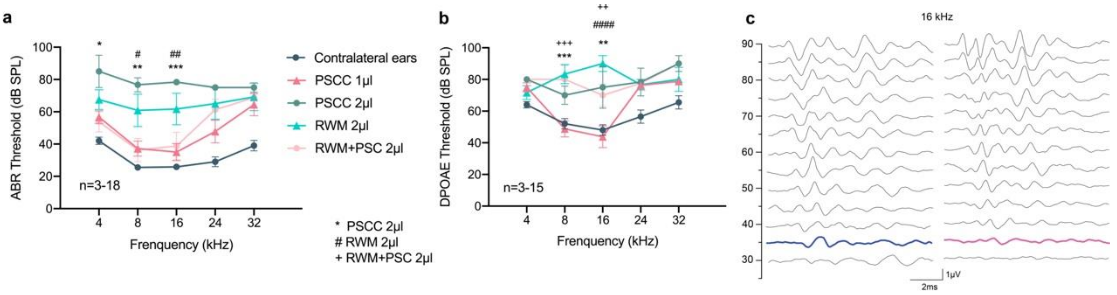

Figure S4. The hearing results for all groups at 2 weeks after the Anc80L65 injection in adult mice. (a) ABR tests showed significant differences between the 1 μL PSCC group and the 2 μL PSCC group at 4, 8, and 16 kHz (*P = 0.0492, 0.0011, 0.0002*) and significant differences between the 1 μL PSCC group and RWM group at 8 and 16 kHz (*P = 0.0308, 0.0087*). (b) DPOAE tests showed significant differences between the 1 μL PSCC group and 2 μL PSCC group at 16 kHz (*P = 0.0031*), significant differences between the 1 μL PSCC group and RWM group at 8 and 16 kHz (*P = 0.0007, <0.0001*), and significant differences between the 1 μL PSCC group and the t-RP group at 8 and 16 kHz (*P = 0.0011, =0.0100*). (c) ABR waveforms from the representative contralateral ear and the ear injected with 1 μL AAV-Anc80L65 vector through the PSCC approach at 16 kHz. The blue trace indicates the threshold of the contralateral ear, while the pink trace indicates the threshold of the injected ear. The scale bar applies to all traces. Two-way ANOVA with Bonferroni correction was performed for multiple comparisons for ABR and DPOAE tests. **p < 0.05; **p < 0.01; ***p < 0.001; ****p < 0.0001.*
